# Gynecologic Oncology and Inclusion of Women Into the Surgical Workforce: The Canary in This Coal Mine

**DOI:** 10.3389/fonc.2022.789910

**Published:** 2022-04-06

**Authors:** Linda J. Hong, Lisa Rubinsak, Michelle F. Benoit, Deanna Teoh, Uma Chandavarkar, Amy Brockmeyer, Erin Stevens, Yevgeniya Ioffe, Sarah M. Temkin

**Affiliations:** ^1^ Division of Gynecologic Oncology, Department of Gynecology and Obstetrics, Loma Linda University School of Medicine, Loma Linda, CA, United States; ^2^ Division of Gynecologic Oncology, Department of Oncology, Karmanos Cancer Institute, Wayne State University, Detroit, MI, United States; ^3^ Division of Gynecologic Oncology, Department of Obstetrics and Gynecology, Kaiser Permanente Washington, Seattle, WA, United States; ^4^ Division of Gynecologic Oncology, Department of Obstetrics, Gynecology and Women’s Health, University of Minnesota, Minneapolis, MN, United States; ^5^ Department of Oncology, Sutter Medical Group, Sacramento, CA, United States; ^6^ Division of Gynecologic Oncology and Gynecology, Department of Surgery, Virginia Mason Medical Center, Seattle, WA, United States; ^7^ Division of Gynecologic Oncology, Department of Obstetrics and Gynecology, Prevea Health, Green Bay, WI, United States; ^8^ Independent Researcher, Washington, DC, United States

**Keywords:** gender discrimination, women in medicine (WIM), women in surgery, gynecologic oncologists, inclusion, microaggressions, bullying

## Abstract

**Objective:**

Women make up a majority of the gynecologic oncology workforce. Increasing the numbers of women in leadership has been proposed as a path towards professional gender equity. This study examined whether leadership gender and departmental infrastructure impact the work environment for women gynecologic oncologists.

**Methods:**

Members of a 472-member private Facebook group “Women of Gynecologic Oncology” (WGO) who self-identified as women gynecologic oncologists provided demographics, practice infrastructure, personal experience with workplace bullying, gender discrimination, microaggressions using a REDcap survey platform.

**Results:**

Of 250 (53%) respondents to this survey, most were younger than age 50 years (93.6%); White (82.2%) and non-Hispanic (94.3%); married (84.7%); and parenting (75.2%). Practice environments included academic (n=152, 61.0%), hospital employed (n=57, 22.9%), and private practice (n=31, 12.4%), and 89.9% supervised trainees. A significant percent of respondents had experienced bullying (52.8%), gender discrimination (57%) and microaggressions (83%). Age, race, ethnicity, practice setting, or mentorship were not statistically significantly associated with these experiences. Reported perpetrators were varied and included colleagues (84%), patients (44%), staff (41%), administrators (18%), and trainees (16%). Prevalence of bullying (55.0 vs 47.7%, p=0.33), gender discrimination (59.1 vs 52.3%, p=0.33) and microaggressions (83.3 vs 83.0%, p=1.00) were similar irrespective of departmental leadership gender.

**Conclusions:**

Women gynecologic oncologists report a high prevalence of workplace bullying, gender discrimination and microaggressions regardless of the gender of their immediate leadership. Proactive and deliberate structural interventions to improve the work environment for surgeons who are women are urgently needed.

## Introduction

Gender equity remains a critical issue for physicians who are surgeons (1). Although women have comprised more than half of medical students since 2003, women have remained less likely to enter and remain within surgical specialties (1, 2). Gynecology is a notable exception. By the 1990’s half of trainees in obstetrics and gynecology were women. More than half of physicians practicing obstetrics and gynecology have been women since 2012; currently 59% of practicing gynecologists are women and 83% of trainees are women (3–5). Gynecologic oncology is a unique gynecologic subspecialty that requires competency in radical pelvic surgery including upper abdominal, bowel and bladder procedures. Similar to obstetrics and gynecology as a whole, increasing numbers of women have entered this field. In 2020, more than half of gynecologic oncologists and 70% of trainees self-identified as female (6).

Despite the majority of gynecologic oncologists identifying as women, recent literature supports the persistence of significant gender disparities in attaining leadership (7), a gender wage gap that is unexplained by experience or skill (6, 8), and high rates of perceived discrimination (9, 10). In a recent survey of gynecologic oncologists, 64% of female respondents endorsed gender discrimination in training or practice (10). Women remain under-represented in leadership relevant to gynecologic oncology including division director and department chair, but the presence of women in leadership roles has been previously associated with positive work environments within the subspecialty (7).

The objective of this study was to assess whether leadership gender and practice infrastructure are associated with the prevalence of bullying, gender discrimination, and microaggressions among surgeons who are women.

## Methods

A sample of women gynecologic oncologists was recruited from the Facebook group, the “Women of Gynecologic Oncology” (WGO), an online Facebook community established August 18, 2017. At the time of the study, there were 472 members of this group of an estimated 1126 gynecologic oncologists in the United States (77.6% of the women workforce in gynecologic oncology) (6). The group is active with 453 members who signed on the month of the survey with 98 new posts, 972 comments, and 1,956 reactions from members during this time period. A link to an anonymous, secure survey administered *via* Research Electronic Data Capture (REDCap) (11) was posted on the WGO Facebook page and remained active between 7/20/2020 and 8/19/2020. The survey was optimized for use on computers as well as mobile devices. Written informed consent was included in the survey. The study was granted exemption status by the Institutional Review Boards at Loma Linda University Health.

The survey included 69 multiple choice items with branching logic. Demographic data collected included age, race/ethnicity, marital status, parenting status, geographic location, and years in practice. Practice setting was categorized as academic, hospital employed, private practice, HMO, military or other. Respondents were asked whether they oversee trainees, the department to which they report, their leadership gender and sub-specialty and whether they have a formal assigned mentor. Satisfaction with parental leave and whether breast-feeding goals had been met were queried to assess work-life balance.

Bullying was defined within the survey for respondents as “the use of negative and aggressive interpersonal behaviors to intimidate and dominate others. Bullying behaviors often are persistent and repeated. Examples include humiliation, insults, threats, coercion, isolation, and overwork—sometimes involving repetitive or meaningless tasks. Discrimination was defined as “negatively charged, differential treatment based on one’s personal characteristics or attributes, including, but not limited to, gender, race, religion, sexual orientation, culture, ethnicity, disability, or age.” Microaggressions were evaluated using questions felt to be most relevant to a surgical practice from the validated survey the “sexist mess” (12, 13) and defined as “everyday verbal, nonverbal, and environmental slights, snubs, or insults, whether intentional or unintentional, which communicate hostile, derogatory, or negative messages to target persons”.

Data regarding bullying, harassment, and microaggression including perpetrators and effects on the respondent’s career were captured. Missing values were excluded by line. Descriptive statistics were compiled. Univariate analysis was performed using χ^2^ tests. A multivariate logistic regression model was created to study the association of partner occupation with the respondents’ desire to switch to a less demanding career or specialty while controlling for potential confounders. Variables were chosen based on contextual plausibility and statistical significance on initial univariate analysis. All *p* values were from 2-sided tests, and results were deemed statistically significant at *p* ≤ .05. All analyses were performed using JMP^®^, Version 15. SAS Institute Inc., Cary, NC, 1989-2019.

## Results

Four hundred and fifty three of 472 WGO members logged onto the Facebook group while the survey was active. Of these members 250 (55%) submitted survey responses. Demographics of those respondents are detailed in **Table 1**. Most respondents were younger than age 50 years (93.6%), white (82.2%) and non-Hispanic (94.3%). A majority were married (84.7%) and had children (75.2%). Practice environments are described in **Table 2** and included academic (n=152, 61.0%), private practice (n=31, 12.4%), and hospital employed (n=57, 22.9%), and 89.9% supervised trainees. Most respondents reported within a department of obstetrics and gynecology (77.5%) to male division directors (56.7%) and male department chairs (60%). Only 16.1% of respondents had a formal, assigned mentor.

**Table 1:** Demographics of survey respondents.

Characteristic	N (%)
Age	
30-40 years old	160 (64.0)
41-50 years old	74 (29.6)
>50 years old	16 (6.4)
Race	
Asian	25 (10.1)
Black	5 (2.0)
Native American	1 (0.4)
White	203 (82.2)
Two or more races/Other	13 (5.2)
Ethnicity	
Hispanic/Latina	14 (5.7)
Partner Status	
Married or in a long-term relationship	223 (89.6)
Separated/Divorced or Single	26 (10.4)
Parenting	188 (75.2)
Geographic Region	
Northeast	60 (24.2)
Midwest	52 (21.0)
South	82 (33.1)
West	50 (20.2)
Canada	4 (1.6)
Years in Practice	
Fellow in training	47 (18.7)
1-10	162 (65.1)
11+	40 (16.1)

**Table 2:** Practice characteristics of survey respondents.

Practice Type	
Academic	152 (61.0)
Hospital Employed	57 (22.9)
Private Practice	31 (12.4)
HMO, Military, Other	9 (3.6)
Oversee Trainees	221 (89.1)
Reporting Structure	
OBGYN	193 (77.5)
OBGYN Chair specialty	
General OBGYN	54 (28.4)
Maternal Fetal Medicine	53 (27.9)
Gynecologic Oncology	34 (17.9)
Urogynecology (FPMRS)	30 (15.8)
Reproductive Endocrinology	16 (8.4)
Surgery	20 (8.0)
Other	36 (14.5)
Female Department Chair	88 (40)
Female Division Director	87 (43.3%)
Formal Mentor	40 (16.1%)

The experience of bullying was endorsed by 131 of 248 (52.8%) of respondents, 100 (76.3%) in training and 81 (61.8%) in practice. Gender discrimination was endorsed by 142 of 249 respondents, 112 (78.9%) in training and 92 (64%) in practice. Two hundred and eight (83.5%) respondents endorsed being the target of any microaggression. Experiences included the following: (1) hiding personal life or changed personality to adapt to a work environment (56.6%); (2) pretending to be interested in an activity to feel included in a conversation (41.9%); (3) being told to smile more (30.9%); (4) being told at work to dress a certain way (19.7%); and being told to act more female, nurturing and/or motherly (16.1%). No demographic or practice characteristics, including having an assigned mentor were statistically associated with experiences of bullying, discrimination, and microaggressions.

Division director gender, specialty or department reporting structure were not statistically significantly associated with the experience of bullying, gender discrimination or microaggressions (**Table 3**). Compared to respondents with a male chair, those with a female chair experienced similar rates of bullying (55.0 vs 47.7%, p=0.33); gender discrimination (59.1 vs 52.3%, p=0.33); and microaggressions (83.3 vs 83.0%, p=1.00). Women with male department chairs were more likely to meet breast-feeding goals (81 vs 60.4%, p-0.02). No other significant associations were identified between chair gender and perceived work environment for respondents.

**Table 3:** Association between leadership gender and workplace experience.

	Female GO Division Chair n = 87 (43.3%)	Male GO Division Chair n = 114 (56.7%)	*p* value	Female Department Chair n = 88 (40%)	Male Department Chair n = 132 (60%)	*p* value
Experienced bullying?	47 (54%)	60 (52.6%)	0.84	42 (47.7%)	72 (45%)	0.64
Experienced gender discrimination?	49 (56.3%)	68 (59.7%)	0.64	46 (52.3%)	78 (59.1%)	0.32
Experienced microaggression?	74 (85%)	96 (84.2%)	0.94	73 (83%)	110 (83.3%)	0.94
Insufficient parental leave?	34 (64.2%)	44 (68.8%)	0.6	36 (64.3%)	48 (69.6%)	0.53
Met personal breastfeeding goals?	38 (74.5%)	36 (65.5%)	0.31	29 (60.4%)	51 (81%)	**0.02**
Adequate departmental support?	59 (68.6%)	71 (65.1%)	0.61	52 (61.2%)	87 (69.6%)	0.21
Adequate division support?	71 (84.5%)	82 (74.6%)	0.09	66 (79.5%)	96 (76.8%)	0.64
Excluded from leadership role due to gender?	26 (29.9%)	46 (40.3%)	0.12	27 (30.7%)	52 (39.4%)	0.19

OBG, Obstetrics and Gynecology; GO, Gynecologic Oncology. Bold values statistically significant at p < 0.05 values.

Formal institutional reporting of bullying and discrimination was uncommon (24.4 and 19% respectively). When reported, bullying and discrimination was most commonly reported to a chief or chair (78.1 and 77.8%, respectively) and human resources (18.8% and 29.6%, respectively). The frequency of reporting was not associated with practice environment. Respondents who worked for a woman department chair were more likely to report experiences of bullying (33.3% vs 16.7%, p= 0.04) but not discrimination.

**Figure 1:**
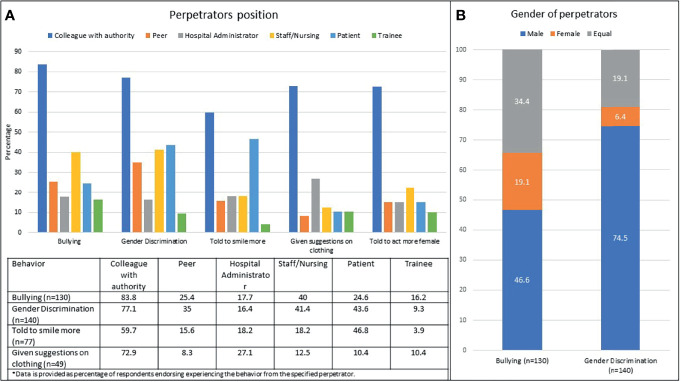
Perpetrators of bullying and discrimination were multiple and varied. **(A)** Positions of the perpetrators; **(B)** Gender of the perpetrators.

Multiple and varied perpetrators of bullying, gender discrimination and microaggression were reported (**Figure 1**). Individuals instigating these behaviors were identified as colleagues with authority (84%), patients (44%), staff (41%), administrators (18%), and trainees (16%). Perpetrators of bullying and discrimination were more commonly male, but significant numbers of respondents reported female instigators and/or an equal amount of bullying and discrimination from men and women.

Survey respondents endorsed that gender had impacted their careers and advancement (**Table 4**). Bullying and gender discrimination led to job changes for 18.3 and 13.6%, respectively, of participants. Nearly half of respondents endorsed exclusion from networking opportunities and over a third reported exclusion from leadership that was perceived to be related to gender. A woman in a leadership role was perceived as having been a barrier to advancement for 32.1% of respondents. Being written up for speaking one’s mind that was perceived to have been tolerated from male colleagues was common. Ten percent of respondents had been the subject of a sham peer review (defined as the abuse of a medical peer review process to attack a doctor for personal or other non-medical reasons). Few respondents (12%) felt that gender negatively impacts their male colleagues.

**Table 4:** Perceived influence of gender on the careers of women gynecologic oncologists.

Outcome	Response n (%)
Excluded from networking opportunities due to gender	106/249 (42.6)
Excluded from a leadership position due to gender	90/249 (36.1)
Written up for speaking your mind in a way that would have been tolerated from a male colleague	82/249 (32.9)
Had a woman be a barrier to advancement	80/249 (32.1)
Changed jobs because of bullying	24/131 (18.3)
Changed jobs because of gender discrimination	19/141 (13.6)
Been involved in a sham peer review, defined as the abuse of a medical peer review process to attack a doctor for personal or other non-medical reasons?	24/249 (9.6)
Observed gender negatively impacting the careers of your male colleagues	30/249 (12.0)

## Discussion

The findings of the current study confirm that gender-based discrimination remains a common experience for gynecologic oncologists who are women; that reporting of these experiences is uncommon; and provides evidence that this environment negatively impacts the career trajectories of women, leading to lost opportunities and job changes. By capturing perceived bullying and microaggressions, this study demonstrates the pervasiveness of hostility related to gender for women, despite the large numbers of women practicing within this surgical specialty. A previous, recent survey of gynecologic oncologists demonstrated that 71% of women and 51% of men reported experiencing sexual harassment in training or in practice. Few targets (14.5%) reported their experience. Female respondents were more likely to have had the experience impact their career advancement or compensation (10). Prior reports have linked experiences of gender discrimination and sexual harassment to increased reports of burnout, job changes and career dissatisfaction (9, 10, 14–17).

“Critical mass” theory of the 1970’s proposed that once women represented a third of a professional group, the culture of the group would shift such that they would no longer be perceived as a minority or subordinate (18). However, increasing proportions of women in a surgical work environment does not necessarily correlate to decreased prevalence of gender discrimination or sexual harassment. In a survey of general surgery training programs, increasing proportions of female residents was correlated with program-level rates of gender discrimination (r=0.64; 95% CI, 0.56-0,70) and sexual harassment (r=0.17; 95% CI, 0.06-0.28) (19). In gynecologic oncology experiences of bullying and discrimination persists despite large numbers of women – most recently in the “2020 Society of Gynecologic Oncology State of the Specialty” report, 54% of gynecologic oncologists self-identified as female (6), at rates remarkably similar to those reported in surgical specialties that remain predominated by men. A survey of the members of the American College of Surgeons and Association of Women Surgeons, found that 58% of women and 25% of men experienced sexual harassment in the preceding 12 months (20). In a survey of 927 practicing orthopedic surgeons 81% of women reported having experienced harassment, discrimination or bullying (21).

The professional roles and genders of the perpetrators of bullying and harassment reported in this study were multiple and varied. Significant numbers of respondents identified peers, staff/nursing, administrators, patients and trainees as the source of unprofessional behaviors. Nurses were the most commonly identified perpetrators of harassment in a recent survey of 270 general surgery trainees in which 48% percent of female and 22% of male respondents reported being harassed by nursing staff (22). Another recent survey of trainees identified the most common sources of discrimination as patients and nurses and that events occurred more often in the emergency and operating rooms (23). A qualitative study of 30 women surgeons described frequent workplace conflict with a non-physician and those interactions frequently resulting in formal reporting of the surgeon. Common impact themes, including personal (emotional and physical), professional, and patient safety were identified (24).

Promoting more women into leadership has often been proposed as a means with to reduce gender inequities in medicine and surgery (7, 25, 26). Yet despite large numbers of women in divisions and departments led by women, our study did not find an association between leadership gender and the experiences of bullying, gender discrimination or microaggressions. The only significant associations with women whose department chairs were men were more likely to reach breast feeding goals, while women were more likely to report bullying when working for a female department chair. These findings support the concept that organizational and institutional acknowledgement, accountability and education for leaders is necessary in order for culture change to be successful in traditionally hierarchical academic medical centers (27).

Our study confirmed that reporting of bullying and discrimination is uncommon among targets of sexual harassment (1, 10, 15, 22). Organizational prioritization of reporting and investigating harassment is necessary to create the transparency required to address the culture that allows bullying, harassment and microaggressions. In 2017 an anonymous reporting system was developed and implemented at the Mayo Clinic demonstrating the feasibility of institutional accountability. All allegations of sexual harassment are duly investigated by an HR representative in conjunction with the legal department. Over the first 2 years, 153 allegations were made and 88 (57.5%) substantiated. Of these, 71 (80.7%) included inappropriate comments and/or unwelcome sexual advances, 22 (25%) unwanted touch or physical contact, and 16 (18.2%) virtual or electronic harassment (e.g., email, messenger, text). Investigations resulted in 31 employees receiving formal coaching, 22 receiving written warnings, and 35 terminations or resignations (28). An improved culture has not yet been documented; however, the perception of fairness and justice are important foundations for eliminating cultures that allow harassment (1, 29, 30).

The negative effects of bullying and harassment in the workplace extend to surgeons of any gender. Observing discriminatory behavior is detrimental to the well-being of those exposed (15, 17, 31). Engaging bystanders may also be a key component to changing the culture of surgery as bystanders interventions are an effective evidence-based tool that can be used to combat discrimination (32, 33).

This study was designed to provide information about the effect of gender on work environment among women who are surgeons in a specialty with a majority women workforce. As such the prevalence and impact of bullying, discrimination and microaggressions among men were not captured. The results presented in this manuscript are limited by the biases inherent in a survey design and the convenience sample used to obtain responses. Gender bias in medicine is often insidious (34, 35) and our results may reflect recall bias. Gynecologic oncologists who experience a negative work environment may be more likely to actively participate in a social media support group. Our results may have undercounted the experiences of bullying and harassment across a woman’s career as our survey respondents were primarily young, therefor may not yet have experienced the accumulation of events reported by midcareer and senior surgeons who are women (36). Similarly, most participants were White, and may not have experienced the amplified experiences bullying and harassment reported by physicians from historically underrepresented in medicine communities who are also women (21, 37, 38). In addition, we were unable to assess the leadership gender within the broader context of the organization which may have contributed to our findings. The strengths of this study, however, include the large proportion of gynecologic oncologists who are women who participate actively in this social media group, the WGO and the high response rate (over half of active participants).

An urgent need to correct gender-based hostility within the surgical work environments exists. Inclusion of women into a specialty and into leadership positions is insufficient to create belong within surgical workplaces. Department and institutional leaders and mentors need to actively help women navigate unprofessional behavior, gender bias, and exclusion at work. Although gynecology was the first surgical specialty to attract a majority women workforce, increasing numbers of women are entering all surgical specialties (1, 39). With an increasing number of women in the surgical workforce, attention to creating spaces where all surgeons can thrive should be prioritized by all healthcare systems. Further studies are needed to address how the systemic change can occur and be standardized in institutions.

## Data Availability Statement

The raw data supporting the conclusions of this article will be made available by the authors, without undue reservation.

## Ethics Statement

The studies involving human participants were reviewed and approved by Loma Linda University Institutional Review Board. Written informed consent for participation was not required for this study in accordance with the national legislation and the institutional requirements.

## Author Contributions

LH, ST, MB, and LR contributed to conception and design of the study. LH, DT, MB, and ST designed the survey. LR organized the database. LR performed the statistical analysis. ST wrote the first draft of the manuscript. All authors contributed to manuscript revision, read, and approved the submitted version.

## Conflict of Interest

The authors declare that the research was conducted in the absence of any commercial or financial relationships that could be construed as a potential conflict of interest.

## Publisher’s Note

All claims expressed in this article are solely those of the authors and do not necessarily represent those of their affiliated organizations, or those of the publisher, the editors and the reviewers. Any product that may be evaluated in this article, or claim that may be made by its manufacturer, is not guaranteed or endorsed by the publisher.
